# Unveiling the Significance of Peroxiredoxin 6 in Central Nervous System Disorders

**DOI:** 10.3390/antiox13040449

**Published:** 2024-04-10

**Authors:** Min Xue, Xiaojie Huang, Tong Zhu, Lijun Zhang, Hao Yang, Yuxian Shen, Lijie Feng

**Affiliations:** 1 School of Basic Medical Sciences, Anhui Medical University, Hefei 230032, China; xuemin@ahmu.edu.cn (M.X.); huangxj@njfu.edu.cn (X.H.); 2023510042@ahmu.edu.cn (T.Z.); 2346010004@stu.ahmu.edu.cn (L.Z.); 2245010022@stu.ahmu.edu.cn (H.Y.); shenyx@ahmu.edu.cn (Y.S.); 2 Institute of Biopharmaceuticals, Anhui Medical University, Hefei 230032, China

**Keywords:** Prdx6, glutathione peroxidase, phospholipase A2, central nervous system, neuroinflammation, neurodegeneration

## Abstract

Peroxiredoxin 6 (Prdx6), a unique 1-Cys member of the peroxiredoxin family, exhibits peroxidase activity, phospholipase activity, and lysophosphatidylcholine acyltransferase (LPCAT) activity. Prdx6 has been known to be an important enzyme for the maintenance of lipid peroxidation repair, cellular metabolism, inflammatory signaling, and antioxidant damage. Growing research has demonstrated that the altered activity of this enzyme is linked with various pathological processes including central nervous system (CNS) disorders. This review discusses the distinctive structure, enzyme activity, and function of Prdx6 in different CNS disorders, as well as emphasizing the significance of Prdx6 in neurological disorders.

## 1. Introduction

Peroxiredoxins (Prdx) are a ubiquitous family of highly conserved antioxidant enzymes featuring a cysteine (cys) residue involved in peroxide reduction. In Homo sapiens, to date, six isoforms of Prdx (Prdx1–Prdx6) have been reported, which are categorized into three subgroups based on the position and number of cysteine residues participating in catalysis: intermolecular (typical 2-Cys, Prdx1–4), intramolecular (atypical 2-Cys, Prdx5) disulfide bonds, and noncovalent interactions (1-Cys, Prdx6). Distinct from other family members, Prdx6 is a unique 1-Cys peroxidase and does not utilize thioredoxin as a reducing agent [1,2]. Prdx6 is a multifunctional enzyme with peroxidase activity, antioxidant-acting acidic calcium-independent phospholipase A2 (aiPLA2) activity, and lysophosphatidylcholine acyltransferase (LPCAT) activity [3,4,5]. These unique enzyme activities make it interesting to explore the physiological and pathological functions of Prdx6 in different diseases. Over the past several years, Prdx6 has been extensively investigated in brain diseases, such as Alzheimer’s disease (AD) and Parkinson’s disease (PD) [6,7]. Nevertheless, the underlying mechanisms of action for Prdx6 in neurological disorders have largely remained elusive. Therefore, this review seeks to unveil the significance of Prdx6 in CNS disorders, which will contribute to a greater understanding about the potential value of Prdx6 as a new therapeutic target for neurological diseases.

## 2. Structure of Prdx6

**Figure 1:**
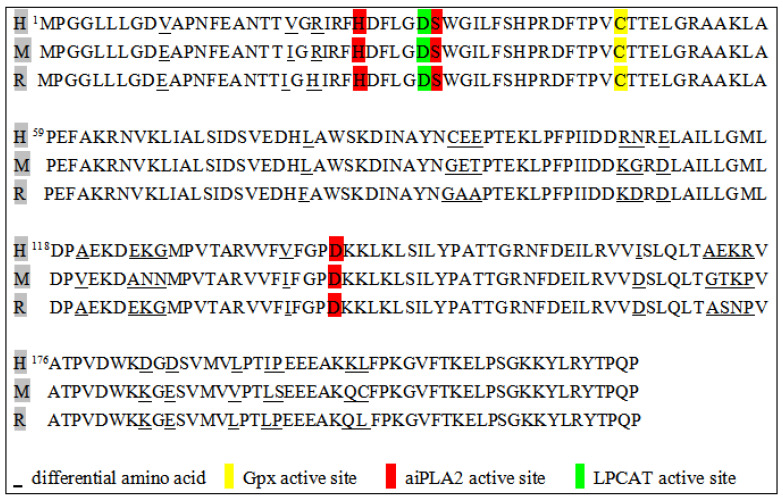
Prdx6 amino acid sequence for a human (H), mouse (M), and rat (R).

Human Prdx6 protein consists of 224 amino acids and is encoded by an 11,542 base-pair gene with five exons. It is located on human chromosome 1, as verified by the cloning and sequencing of the Prdx6 cDNA. It has a relative molecular weight of 25–29 kDa on SDS/PAGE and an isoelectric point (pI) of 5.1, which varies depending on the oxidation and phosphorylation status of the protein [8,9]. The comparison of Prdx6 proteins among mammals (humans, mice, and rats) reveals an 95% identity in amino acid sequences (Figure 1). Compared with other proteins in the Prdx family, Prdx6 has a single conserved cysteine residue, known as the 1-Cys Prdx subgroup, and an additional Cys residue presented in the N-terminus (C47) of humans and mice, whereas this residue is absent in rats and bovines [10]. The secondary structure of Prdx6 contains a typical thioredoxin fold, consisting of approximately 80 amino acids arranged as four inverted β-sheets between two α-helices [11,12]. Furthermore, a low pH results in the conformational transformation of Prdx6 into high-order oligomers, and oligomer formation underlies the resistance of human Prdx6 at the lysosomal pH and high temperatures [13]. Some studies have shown that Prdx6 is a pH-dependent enzyme, and this pH specificity is attributed to the differential substrate preference at different pH values, such as peroxided phospholipids at neutral pH and reduced phospholipids at acidic pH [14]. Prdx6 regulates oxidative stress, and this function has been identified in the cerebral cortex and hippocampus of young and old mice [15]. Evidence suggests that Prdx6 serves as a rheostat in regulating cell physiology by scavenging reactive oxygen species (ROS) to optimize gene regulation. Prdx6 ameliorates ROS-based oxidative damage and NF-κB-mediated aberrant signaling in human cortical neuronal cells, HCN-2, and mouse hippocampal cells, HT22. Therefore, Prdx6 expression is critical for the protection of neuronal cells from oxidative stress-evoked damage [16]. In a word, the function of Prdx6 is closely related to its structure and enzyme activity.

## 3. Enzyme Activities and Function of Prdx6

Prdx6 can bind and reduce phospholipid hydroperoxides; this process involves peroxidase activity. The peroxidase activity in Prdx6 is associated with distinct active sites (C47, R132, H39) [8]. Prdx6 expresses only a conserved Cys and uses glutathione (GSH) and GSH S-transferase (GST) for the reduction and resolution of its oxidized peroxidatic Cys [17]. The activity of phospholipid hydroperoxidase GSH peroxidase (PHGPx) is mediated by the catalytic cysteine at position 47 (C47). The mutation of cysteine to serine (C47S) in Prdx6 can eliminate its ability to reduce hydroperoxides [18,19]. Prdx6, as a peroxidase, has substrate specificity, and its substrates include H_2_O_2_, short-chain hydroperoxides, and phospholipid hydroperoxides [20,21]. Prdx6 binds to oxidized lipid substrates (oxidized membrane phospholipids) and reduces the generation of phospholipid hydroperoxides induced by oxidative stress [22,23]. Also, Prdx6 interacts with phospholemman in a glutathione-dependent manner and depalmitoylates phospholemman via reactive thiol [24]. The peroxidase activity of Prdx6 is a conformation-driven process based on the redox state, which essentially involves monomer-dimer transition [9].

The enzyme activity of Prdx6, Ca^2+^-independent intracellular phospholipase A2 (aiPLA2) activity, was discovered successively in rat and bovine lung tissues. The aiPLA2 activity plays important roles in the synthesis and phospholipid conversion of the lung surfactant. The aiPLA2 activity has been confirmed in the deduced amino acids from the nucleotide sequences of clones isolated from a cDNA library of human adult myeloid cells [17,25]. The catalytic triad (S32, H26, and D140) of phospholipase activity resides on the surface of the Prdx6 protein, which plays a pivotal role in the reduction of oxidized phospholipids and cell-membrane remodeling [26]. Research has indicated that the mutations in the His26 and Ser32 of Prdx6 lead to the loss of the ability to bind phospholipids and the aiPLA2 activity. Nevertheless, the Asp140 mutation causes a loss of aiPLA2 activity but does not affect binding to phospholipids [10]. Prdx6 can exert aiPLA2 activity in the cytoplasm at a neutral pH and in the lumen of acidic lysosomes. The aiPLA2 activity is highest when phosphatidylcholine is used as the substrate, but gradually decreased when phosphatidylethanolamine, phosphatidylglycerol, inositol, and serine are the substrates [27]. The aiPLA2 catalyzes the hydrolysis of the sn-2 fatty acyl ester bond of glycerophospholipids to produce free fatty acids and lysophospholipids. In addition, it has an essential physiological role in the repair of peroxidized cell membranes and the activation of NADPH oxidase 2 (Nox2) [10,28,29].

Initially, it was thought that Prdx6 is a bifunctional enzyme, but recent studies reported that Prdx6 also has LPCAT activity. The current investigation provides evidence that Prdx6 exhibits acyltransferase activity, a function dependent on the presence of the amino acid D31 site involved in lipid metabolism [5]. The LPCAT activity is relatively specific for lysophosphatidylcholine and palmitoyl-CoA. There is no release of intermediates when LPCAT activity is combined with the aiPLA2 activity of Prdx6 [30]. In addition, LPCAT activity is a critical component of the phospholipid remodeling pathway. The reduction of phospholipid hydroperoxides depends on the synergistic activation of the LPCAT and aiPLA2 activities of Prdx6. Therefore, it can play a role in the lipid synthesis remodeling pathway and act as an integral enzyme to repair peroxidized cell-membrane phospholipids [31].

Beyond its enzymatic functions, research has explored the impact of how Prdx6 interacts with other proteins or drugs on pathological processes and homeostasis. In non-alcoholic steatohepatitis (NASH), the interaction between Miz1 and Prdx6 in hepatocytes is crucial. A loss in Miz1 leads to the inhibition of mitophagy mediated by Prdx6 and triggers the production of pro-inflammatory cytokines by hepatic macrophages [32]. Liu et al. demonstrated that the nuclear phosphoprotein (NPM) regulates the expression of Prdx6 and affects the level and distribution of ROS, particularly in tumor cells [33]. Daverey and Agrawal showed that curcumin protects astrocytes from oxidative stress by reducing astrocyte GFAP, decreasing waveform proteins, and inhibiting Prdx6 expression [34]. Another study reported that Bmal1 and Nrf2 could directly regulate the transcription of Prdx6 in human lens/lens epithelial cells (hLECs) by binding to the E-box element and ARE sites, respectively, which cooperated to activate the Prdx6 transcription in hLECs and facilitate the antioxidant defense to maintain redox homeostasis [35]. A Nrf2 activator, Sulforaphane (SFN), reactivates cellular antioxidant defense by inducing Prdx6 activity to influence the intracellular homeostasis [36,37].

## 4. Prdx6 Expression in the CNS

The Prdx6 protein is originally isolated from the bovine ciliary body [2]. In mammalians, Prdx6 is expressed in almost all organs, especially in the lungs, brain, kidneys, and testes [38,39]. In the CNS, the Prdx6 protein is expressed in olfactory areas, the cortex, the hippocampus, thalamic areas, the hypothalamus, the brainstem, the cerebellum, and the spinal cord [40]. At the cellular level, Prdx6 is mainly expressed in astrocytes and not in other glial cells [6]. The intracellular expression of Prdx6 is localized in the cytoplasm and acidic organelles, such as lysosomes and lysosome-associated organelles [4]. Prdx6 is expressed during brain development and increases after birth [41]. Therefore, the lack of oxidative-damage protection in early development may be related to the low expression of Prdx6. Prdx6 remains in a dormant state in a typical brain, while in some pathological states, such as glioma and AD, Prdx6 is selectively upregulated in astrocytes [42,43]. It is likely that the expression of Prdx6 in activated astrocytes may contribute to initiating oxidative stress. In experimental autoimmune encephalomyelitis (EAE) mice and multiple sclerosis (MS) patients, the expression of Prdx6 is markedly increased in spinal cord astrocytes, which may be related to high levels of nitric oxide (NO) and superoxide after EAE and MS [44].

## 5. Prdx6 and CNS Diseases

### 5.1. Alzheimer’s Disease (AD)

AD is a progressive neurodegenerative disease characterized by hyperphosphorylated tau and abnormal beta-amyloid deposition, as well as neuronal degeneration [45]. In the brains of AD patients, Prdx6 was significantly increased in astrocytes in both white and gray matter in the midfrontal cortex, cingulate, hippocampus, and amygdala, and astrocytes with a high expression of Prdx6 participated in the detoxification of diffuse plaque [43] (Table 1). Furthermore, significantly elevated Prdx6 expression in astrocytes was identified exclusively in AD patients, not in controls, which was related to disease-associated glial cell activation in AD [46]. However, a proteomic study found that compared to typical sporadic AD, the levels of Prdx6 in amyloid plaques in rapidly progressing AD (rpAD) was significantly reduced [47]. In addition, there is evidence pointing to a decrease in both Prdx6 mRNA and protein levels in Aβ_1–42_ (amyloid beta 1–42 peptide)-induced AD rats [48]. However, there was no change in the expression levels of Prdx6 between the 3xTg AD mice and the control group [49], which is consistent with the study in the postmortem brain tissue of AD patients [50]. The heterogeneity of Prdx6 expression may be related to different tissues, and in animal models of AD, it may be related to differences in the modeling methods of AD (Table 2).

**Figure 2:**
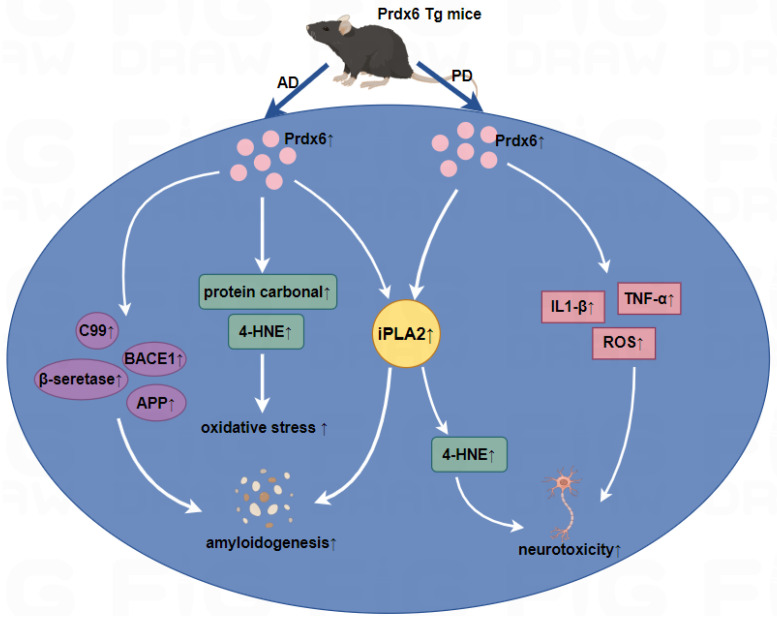
Roles of Prdx6 in Aβ1–42-infused AD mice and MPTP-infused PD mice.

Oxidative stress has been implicated in the pathogenesis of AD patients, and AD brains exhibited an increased thiol oxidation state of Prdx6. The increased expression of the Prdx6 protein in AD was closely related to the degree of oxidative stress [51]. According to reports, aiPLA2 plays a crucial role in the Aβ_1–42_-mediated disturbance of mitochondrial function and oxidative stress in astrocytes [52], which provides new insights into the mechanistic details of Prdx6 in AD. In a correlation study between tumors and AD, the activity of the aiPLA2 of Prdx6 was inhibited through γ-secretase, consequently suppressing the development of lung tumors in AD patients and transgenic mice with mutant presenilin 2 [53]. In Prdx6 transgenic mice infused with Aβ_1–42_, there was an elevation in aiPLA2 activity causing worse memory impairment compared to Aβ_1–42_-infused C57BL/6 mice. The increased aiPLA2 could be involved with the progression of AD. Moreover, Aβ_1–42_-infused Prdx6 transgenic mice exhibited a significant increase in β-secretase activity, protein carbonyl, and 4-HNE levels to increase amyloidogenesis. These data demonstrated that the overexpression of Prdx6 in AD may promote amyloidogenesis and oxidative stress, thereby expediting the progression of AD (Figure 2) [54]. However, increased Prdx6 levels induced by thiacremonone can improve memory dysfunction by interfering with oxidative stress and amyloidogenesis in Aβ_1–42_/H_2_O_2_-induced cultured neuronal cells and amyloid precursor protein/presenilin1 (APP/PS1) transgenic AD mice [55]. There was an upregulation of Prdx6 in astrocytes in P301S transgenic mice, and these astrocytes were infiltrated in the area with a large amount of hyperphosphorylated tau protein and neuron loss, which indicates that Prdx6 may play neuroprotective roles against tau toxicity [56]. Prdx6 was identified as a vital factor regulating astrocyte responses to Aβ plaques. The upregulation of Prdx6 attenuates Aβ pathology and may contribute to AD treatment in the hemizygous knock-in of Prdx6 in APP_swe_/PS1_dE9_ AD transgenic mice, which promotes the selective induction and penetration of astrocytes in Aβ plaques and promotes microglia phagocytic activation [6]. Prdx6 can protect rat PC12 cells from neurotoxicity and resist the oxidative burst in the microglial cell line BV2 stimulated by amyloids [57,58]. In addition, Prdx6 inhibits neurogenesis in neural precursor cells through the TLR4-dependent downregulation of WD-repeat- and FYVE-domain-containing protein 1 (WDFY1) [7]. Although the role of Prdx6 in AD has been extensively investigated, Prdx6 plays different roles in different AD models. Prdx6 can play a protective role in some AD models, such as P301S and APPswe/PS1_dE9_ transgenic mice, and may also accelerate the progression of AD, such as Aβ_1–42_-infused Prdx6 transgenic mice.

**Table 1:** Prdx6 in different AD patients.

Tissues	Species	Expression Level	Expression Tissue	Function	Reference
Postmortem brain	Human	↑	midfrontal cortex, cingulate, hippocampus, and amygdala	Prdx6 plays an anti-oxidant role in AD	[43]
Postmortem brain (female)	Human	↑	superior frontal gyrus	N/A	[46]
rpAD postmortem brain	Human	↓	hippocampus	N/A	[47]
Postmortem brain	Human	no change	frontal cortex and cerebellum	Prdx6 did not show significant changes in the brains of AD patients and possibly has no critical role in cellular defense against oxidative stress.	[50]
Postmortem brain	Human	↑	hippocampus	The increased expression of Prdx6 in AD was closely related to the degree of oxidative stress.	[51]

↑, upregulation; ↓, downregulation; rpAD, rapidly progressive AD; N/A, not applicable.

**Table 2:** Prdx6 in different AD models.

Tissues	Species	Expression Level	Expression Tissue	Function	Reference
APP/PS1 Prdx6 Tg female mice	Mice	↑	cortex and hippocampus	The upregulation of Prdx6 in AD mice can attenuate Aβ pathology.	[6]
3xTg mice	Mice	no change	hippocampus	Prdx6 was not associated with cumulative oxidative stress in animal models of neurodegenerative disease.	[49]
PS2 (N141I) Tg mice	Mice	↓	lung	The PS2 mutation inhibits the aiPLA2 activity of Prdx6 through the γ-secretase cleavage mechanism to suppress lung-tumor development.	[53]
Aβ_1–42_-infused Prdx6 transgenic mice	Mice	↑	cortex and hippocampus	The overexpression of Prdx6 in AD mice promotes amyloidosis and increases oxidative stress, thereby expediting the progression of AD.	[54]
Tau (P301S) Tg mice	Mice	↑	anterior horn of the spinal cord	Prdx6 functions as a neuroprotective mechanism against tau toxicity.	[56]
Aβ_25–35_-treated BV2 cells	Mice	↑	N/A	Prdx6 is protective against oxidative stress in microglia and synergistically maintains the transition to a chronic neuroinflammatory phenotype, reinforcing the role of Prdx6 in AD	[57]
Aβ_1–42_-infused rat	Rat	↑	hippocampus	N/A	[48]
Aβ_1–42_-induced rat primary neuron	Rat	↓	N/A	Thiacremonone influences Prdx6 expression levels and oxidative stress, thereby protecting against amyloidosis and memory dysfunction and inhibiting the development and progression of AD.	[55]
Aβ_25–35_-treated rat PC12 cells	Rat	↑	N/A	Prdx6 can slow the progression of AD and limit the extent of AD-induced neuronal cell death.	[58]

↑, upregulation; ↓, downregulation; APP, β-amyloid precursor protein; PS1, presenilin 1; N/A, not applicable; Tg, transgenic; PS2, presenilin 2.

Left panel: In Aβ_1–42_-infused Prdx6 transgenic mice, the overexpression of Prdx6 could increase iPLA2 activity and oxidative stress in astrocytes, and increase the β-secretase activity and the expression of APP, BACE1, resulting in increased Aβ aggregation, then accelerate the development of AD. Right panel: In the MPTP mice model, the iPLA2 activity of Prdx6 was upregulated followed by an increase in the level of ROS and 4-HNE, and iPLA2 activity induces astrocytic activation and leads to the increased secretion of proinflammatory cytokines such as TNF-α and IL1-β, resulting in dopaminergic neurotoxicity. ↑, upregulation.

### 5.2. Parkinson’s Disease (PD)

PD is an age-related, progressive neurodegenerative disorder characterized by bradykinesia, resting tremor, muscle rigidity, and postural abnormalities [59]. The predominant molecular pathogenesis of PD includes the misfolding and aggregation of alpha-synuclein, mitochondrial dysfunction, impaired protein clearance, neuroinflammation, and oxidative stress [60]. Postmortem brain tissue of PD patients showed that Prdx6 was upregulated in the grey matter and white matter of the frontal and cingulated cortices [61,62] (Table 3). Proteomics analysis using Parkin^−/−^ PD mice revealed a decrease in Prdx6 protein levels [63] (Table 4). aiPLA2 is involved in the development of PD induced by 1-methyl-4-phenyl-1,2,3,6-tetrahydropyridine (MPTP). The aiPLA2 inhibitor quinacrine has a protective effect against MPTP- and 6-hydroxydopamine (6-OHDA)-induced neurotoxicity in mice [64]. In addition, the aiPLA2 activity of Prdx6 is a critical crosstalk between neurons and glial cells in the nigrostriatal dopaminergic neuronal system. The aiPLA2 activity of Prdx6 was increased after MPTP administration in the Prdx6 transgenic mice and upregulated aiPLA2 was accompanied with increased ROS and 4-HNE levels, which results in a greater loss of dopaminergic neurons and increased behavioral damage. And, in MPP+-treated primary astrocytes from Prdx6 transgenic mice, aiPLA2 activity and the release of neurotoxic products as well as reactive oxygen species were increased in MPP+-treated primary astrocytes from Prdx6 transgenic mice to trigger dopaminergic neurotoxicity. These findings demonstrated that the iPLA2 activity of Prdx6 is associated with the progression of PD (Figure 2) [65,66]. In addition, a study showed that Pink1-Parkin-mediated mitochondrial autophagy is ROS-dependent, and Prdx6 is recruited to mitochondria after carbonyl cyanide m-chlorophenyl hydrazone (CCCP) treatment to balance excess ROS, thereby protecting cells from death due to excessive mitochondrial phagocytosis. This suggested that Prdx6 is a key factor in the initial step of mitochondrial clearance and is upstream of the PINK1-Parkin pathway [67]. To sum up, Prdx6 is closely related to the pathogenesis of PD [68], and understanding the enzyme activity of Prdx6 is of great significance for the novel therapeutic approach for PD treatment.

### 5.3. Cerebral Ischemia

Ischemic stroke is a major threat to human health worldwide due to its high morbidity, mortality, and disability rates. Although the exact mechanism is still unclear, oxidative stress, apoptosis, and inflammation have been proven to be involved in the pathogenesis of ischemic stroke [69,70]. Prdx6 is an antioxidant protein that plays an important role in ischemic stroke. The transplantation of cerebral endothelial cells (hCMEC/D3) into cerebral ischemia rats may potentially suppress the expression of Prdx6 induced by ischemia injury to control neuroinflammation, which suggests a potential association between Prdx6 and neuroinflammation in cerebral ischemia [71] (Table 5). A research study analyzed the change in cortical protein profile at 24 h and 2 months after hemorrhagic stroke in white pigs and showed that Prdx6 was significantly increased. Therefore, Prdx6 was related to the activation of neuroprotective compensatory mechanisms [72]. However, in endothelial cells overexpressing endothelin-1 (TET-1) mice, middle cerebral artery occlusion (MCAO) for 30 min followed by reperfusion for 7 days increased the expression of Prdx6 around blood vessels in the ipsilateral hippocampus, leading to neuronal apoptosis, glial activation, and blood–brain barrier disruption [73]. A sustained upregulation of Prdx6 expression may protect hippocampal neurons from oxidative stress in a rat model of stroke (localized heat-induced brain injury in the left anterior cortical tectum) [74,75]. Curcumin treatment upregulated the expression of Prdx6 to attenuate neurological deficits and oxidative stress in cerebral ischemia/reperfusion (I/R) rats, suggesting that Prdx6 has a neuroprotective effect against oxidative stress in rats after stroke [76]. Prdx6 was increased with melatonin treatment to protect neuronal cells from ischemic damage and prevent cell death caused by ischemic injury in MCAO model rats [77,78]. The role of the aiPLA2 activity of Prdx6 following OGD/R has been investigated in a BV2 cell lines/CTX-TNA2 cell lines co-culture system, which showed that aiPLA2 induces ROS production in astrocytes via activating the NOX2 and Drp1 related mitochondrial pathways to promote neuroinflammation [79]. In addition, the aiPLA2 activity of Prdx6 was associated with the secretion of neurotoxic inflammatory factors and a high expression of Toll-like receptor 2/4 (TLR2/4) in cerebral ischemia/reperfusion injury. Inhibiting the aiPLA2 activity of Prdx6 decreased the neurologic deficits, cerebral infarction, and inflammatory molecules [80]. In a recent study, a reduction of Prdx6 activity in the MCAO model resulted in increased neuronal apoptosis through the enhancement of the PINK1/PARKIN pathway-mediated mitochondrial autophagy, therefore exacerbating cerebral ischemia-reperfusion injury and apoptosis [81]. Furthermore, 4-hydroxy-benzylalcohol (4-HBA) mediated the upregulation of Prdx6 to protect neurons against cerebral ischemic injury via the PI3K/Akt pathway [82,83]. However, Prdx6 was proven to be released from necrotic brain cells within 12 h after stroke onset, coinciding with the timing of leukocyte infiltration, then initiating destructive immune responses acting as an endogenous ligand for TLR2 and TLR4 [84,85]. Thus, Prdx6 may exhibit dual roles in cerebral ischemia, potentially linked to its divergent functions within and outside the cell. Hence, there is a need to improve our understanding of the role of Prdx6 in cerebral ischemia to provide reliable validation data for cerebral ischemia treatment.

### 5.4. Spinal Cord Injury (SCI)

SCI is a devastating trauma in the CNS, including primary and secondary damages, with pathological changes such as inflammation, hemorrhage, edema, and oxidative stress, leading to motor, sensory, and functional impairment [86,87]. In contusion SCI rats, a downregulation of Prdx6 was observed, accompanied by an upregulation of TNF-α and an inhibition of motor function. However, the transduction of the TNF-α RNAi vector into the spinal cord increased Prdx6 expression, suggesting that TNF-α inhibition may work as a mechanism for improving motor function via the upregulation of Prdx6 in SCI [88]. A recent study demonstrated that low levels of Prdx6 in reperfusion injury led to increased white-matter inflammation and apoptosis in a rat SCI model, implicating a protective role of Prdx6 in spinal cord hypoxic-reperfusion injury. In addition, Prdx6 activity in white matter was regulated by its cellular distribution and possible interactions of Prdx6 with TNF-α and Nrf2. Nrf2 negatively regulated Prdx6 by inhibiting the aiPLA2 activity of Prdx6, which reduces axonal and astrocyte injury [89]. Therefore, Prdx6 provides a new strategy and target for the clinical treatment of SCI in the future.

### 5.5. Traumatic Brain Injury (TBI)

TBI, which is characterized as an intangible wound, triggers a series of intracerebral events, including hypoxia, oxidative stress, necrosis, apoptosis, and chronic inflammation [90,91]. Evidence from a previous study demonstrated that Prdx6 is a physiologically important redox-sensitive antioxidant component in cerebrospinal fluid. The TBI-induced oxidation of Prdx6 and its specific phospholipid peroxidase activity were correlated with trauma prognosis. The recovery of Prdx6 activity in patients 24 h after the onset of TBI was associated with a good prognosis [92,93,94]. Proteomics analysis revealed the Prdx6 expression was decreased in the brain tissue of diffuse TBI patients compared with those with focal TBI [95]. Other evidence suggests that the Prdx6 level is elevated in the peripheral blood of TBI patients; however, no association was identified between the Prdx6 levels and poor neurological prognosis or mortality at 6 months after surgery in TBI patients, indicating that Prdx6 may serve as a diagnostic marker for acute TBI, but its prognostic ability may be limited [96]. An autoimmunoassay indicated that Prdx6 could be used as a target for autoantibodies induced in response to TBI, and there were high Prdx6 levels in the perivascular area based on the immunohistochemical analysis of the rat cerebral cortex. Studies have shown a dramatic increase in Prdx6 during mild to moderate TBI in a rat TBI model, which indicated that Prdx6 may be a candidate marker of acute mild brain injury [97].

### 5.6. Prion Disease

Transmissible spongiform encephalopathies (TSEs), also known as prion diseases, are fatal neurodegenerative diseases caused by protein misfolding, mitochondrial dysfunction, and oxidative stress, leading to the loss of motor control, paralysis, wasting, and eventually death [98,99]. TSE is caused by the conversion of cellular prion protein (PrPC) to its abnormal isoform (PrPSc) [100,101]. Sporadic Creutzfeldt–Jakob disease (sCJD) is one of many prion diseases characterized by the spontaneous formation of misfolded prion proteins in the brain, and the expression of Prdx6 was increased in the frontal cortex of patients with sCJD [102]. The glycoproteome analysis of a brain revealed that the expression of Prdx6 was continuously increased during the late stages of prion infection in mice [49]. The loss of the Prdx6 protein exacerbated the prion disease, which mainly manifested as astrogliosis and an accumulation of proteinase K-resistant PrPSc, while the overexpression of Prdx6 improved cognitive behavior and attenuated prion-related astrocytosis [103]. Studies have shown that Prdx6 is consumed by peroxides produced by prion-induced oxidative stress, which prevents the emergence of prion-related neuropathology in Prdx6 transgenic mice with prion disease. Enhanced quantities of Prdx6 were identified in the brains of prion-infected mice and neuronal cell lines. Simultaneously, the level of PrPC raised with an increase in PrPSc transformation [104]. Overall, although most studies elucidate the elevated levels of Prdx6 in prion diseases, its role remains somewhat controversial, so additional studies are needed to provide insights into the potential value of Prdx6 in prion diseases.

### 5.7. Multiple Sclerosis (MS)

Multiple sclerosis (MS) is a chronic inflammatory and neurodegenerative disease of the CNS. The primary pathological features are immune-cell infiltration, myelin loss, axonal degeneration, and reactive astrocyte proliferation [105,106]. Evidence from experimental autoimmune encephalomyelitis (EAE) mice and MS patients showed increased expression of Prdx6 in the astrocytes of the spinal cord in comparison with the control group, respectively. At the same time, the upregulation of Prdx6 in EAE mice reduced the loss of myelin, MMP9 expression, and microglia activation to prevent brain-barrier destruction and immune-cell infiltration [44]. Serum levels of Prdx6 in patients with MS were higher than that in control patients with amyotrophic lateral sclerosis and spinocerebellar degeneration. In addition, the serum Prdx6 levels were associated with the albumin quotient, which suggested that Prdx6 is associated with blood–brain barrier dysfunction to some extent in MS [107]. Therefore, Prdx6 may play an important role in the regulation of inflammation and the blood–brain barrier in MS and may be a therapeutic target or biomarker for MS.

### 5.8. Amyotrophic Lateral Sclerosis (ALS)

ALS, also known as motor neuron disease, involves the degeneration of upper and lower motor neurons, resulting in muscle weakness and eventual paralysis [108]. Studies have shown that neuroinflammation and redox dysregulation are important contributors to ALS pathogenesis [109,110]. Prdx6 was uniquely upregulated in mouse ALS models, suggesting that Prdx6 may be a defense against SOD1G93A-induced oxidative stress [102]. However, in a study on the spatiotemporal dynamics in ALS mice, the activity of Prdx6 increases as the disease progresses [111]. Studies on Prdx6 in ALS are limited, and further elucidation of the role of Prdx6 in ALS is needed.

### 5.9. Gliomas

A glioma is the most prevalent primary brain tumor in adults and has an extremely unfavorable prognosis and overall survival [112]. A proteomic study of a patient with oligodendroglioma (ODG) which rapidly developed into anagenic oligodendroglioma (AODG) showed a higher expression of the Prdx6 protein in ODG than in AODG, suggesting that Prdx6 is related to the malignant transformation of ODG and can be regarded as a biomarker of ODG progression [113]. Research from glioma patients revealed a positive correlation between the Prdx6 level and increasing grades of gliomas [114,115]. A gene-expression database has been employed to assess Prdx expression across various glioma subtypes and non-tumor brain tissues. The findings reveal a general increase in Prdx6 expression with the malignant grades of brain gliomas. Elevated Prdx6 expression is notably associated with a lower survival rate among patients, underscoring its pro-cancer effects in gliomas [116]. Hence, Prdx6 may play pro-tumor roles in glioma development and could serve as a potential therapeutic target for gliomas.

### 5.10. Epilepsy

Epilepsy is a chronic neurological disease caused by abnormal paroxysmal neuronal discharges, with cognitive impairment and potential risk of dementia [117]. Proteomic profiling showed increased levels of prdx6 in hippocampuses of patients with mesial temporal lobe epilepsy. But, reduced Prdx6 expression was observed in childhood cortical dysplasia (CCD) patients, a common cause of childhood seizures [118]. A similar tendency of prdx6 expression was found in stargazer (stg) mutant mice, which exhibited several neurological disorders including spontaneous absence seizures [119]. Recent studies indicated that Prdx6 upregulation induced by specificity protein 1 (Sp1) in epileptic hippocampuses may act as aiPLA2 rather than GPx, leading to autophagic astroglial degeneration [120]. Furthermore, the increased aiPLA2 activity of Prdx6 may abrogate GPx1-mediated glutamine synthase (GS) preservation and lead to extended seizure duration due to impaired glutamate–glutamine conversion regulated by GS [121]. These studies suggested that Prdx6 plays an important role in the pathogenesis of epilepsy. Our unpublished studies also showed that the Prdx6 protein was increased in epileptic hippocampus astrocytes, and the knockdown of astrocytic prdx6 relieved neuronal damage via regulating connexin-43-mediated hemichannel activity.

## 6. Conclusions

Prdx6 is a multifunctional enzyme that exerts different effects in CNS diseases. Several studies have shown that Prdx6 is associated with oxidative stress, phospholipid homeostasis, and redox balance. At the same time, Prdx6 can exacerbate or attenuate neuronal damage during disease processes. Therefore, Prdx6 may represent a potential therapeutic agent and target for CNS diseases. Under certain conditions, one or more enzymes have been considered to be the contributing factors in particular diseases. The current research on Prdx6 faces the following challenges: first, Prdx6 has multiple enzyme activities, and the specific enzyme involved in different diseases should be considered. Second, Prdx6 plays diverse roles in various CNS diseases, capable of either protecting or causing damage to the nervous system. Therefore, it may play contradictory roles in different models of the same disease, which makes it more difficult and complex to study the function of Prdx6 in CNS disorders. In addition, Prdx6 may function by interacting with other proteins, rather than relying on its enzyme activity. Further studies are needed to identify the Prdx6-interacting proteins and elucidate their regulatory mechanisms.

Although previous studies about the roles of Prdx6 in brain diseases are somewhat controversial, Prdx6 appears to work primarily via Gpx and aiPLA2 activities to reduce oxidative stress. The human CNS, especially the brain, is extremely sensitive to changes in blood oxygen levels, so it is crucial to study CNS diseases that involve oxidative stress, such as acute brain injury and ischemic stroke. Simultaneously, it can be used as a specific indicator for prognosis in brain diseases. Additional studies are still needed to explore whether Prdx6 can be used as a neuroprotective agent in CNS diseases. Importantly, it is essential to understand the possible roles and underlying mechanisms of Prdx6.

Here, we summarized the studies about CNS diseases and Prdx6 that have been identified over the past two decades, hoping to contribute to future research on this topic. However, due to the specificity of the enzyme activity of the Prdx6, there is a gap in our understanding of it, especially the issue of the signal transduction pathway between the diseases and the protein. Therefore, Prdx6 might represent a potential and alternative target for therapeutic intervention in brain diseases.

## Figures and Tables

**Table 3:** Prdx6 in different PD patients.

Tissues	Species	Expression Level	Expression Tissue	Function	Reference
Postmortem brain	Human	↑	gray and white matter	Prdx6 was upregulated in certain cells to respond to oxidative stress in PD.	[62]
HeLa cells with CCCP-induced GFP-Parkin overexpression	Human	↑	N/A	Prdx6 controls ROS homeostasis during the initial phase of PINK1-Parkin-mediated mitotic phagocytosis.	[67]
HEK293 cells with inducible Parkin expression	Human	↓	N/A	Prdx6 is a potential substrate of the Parkin.	[68]

↑, upregulation; ↓, downregulation; N/A, not applicable; CCCP, carbonyl cyanide m-chlorophenyl hydrazone.

**Table 4:** Prdx6 in different PD models.

Tissues	Species	Expression Level	Expression Tissue	Function	Reference
Parkin^−/−^ mice	mice	↓	ventral midbrain	N/A	[63]
MPTP-induced mice and 6-OHDA-induced rats	mice/rats	↑	striatum	aiPLA2 inhibitor QNC protects against 6-OHDA and MPTP-induced dopaminergic neurotoxicity.	[64]
Prdx6 Tg mice in MPTP administration	mice	↑	substantia nigra and striatum	The aiPLA2 activity of Prdx6 was increased after MPTP administration in the Prdx6 transgenic mice, which results in a greater loss of dopaminergic neurons and increased behavioral damage.	[65]

↑, upregulation; ↓, downregulation; Tg, transgenic; MPTP, 1-methyl-4-phenyl-1,2,3,6-tetrahydropyridine; QNC, quinacrine; 6-OHDA, 6-hydroxydopamine; N/A, not applicable.

**Table 5:** Prdx6 in different stroke models.

Models	Species	Expression Level	Expression Tissue	Function	Reference
TET-1 MCAO	mouse	↑	hippocampus	In TET-1 mice perfused for 7 days after 30 min of MCAO, an increased expression of perivascular Prdx6 in the hippocampus may lead to neuronal apoptosis, glial activation, and blood–brain barrier disruption.	[73]
Tlr2^−/−^; Tlr4^−/−^ MCAO	mouse	↑	N/A	Prdx6 promotes neuronal cell death by activating Toll-like receptor 2 (TLR2) and TLR4 and inducing macrophages to express inflammatory cytokines, including IL-23.	[80]
MCAO	rat	↑	N/A	The MCAO model induced an abnormal increased expression of Prdx6, but after human brain endothelial cell transplantation, Prdx6 levels in the MCAO models decreased.	[71]
Stroke with heat-induced brain injury in the left anterior cortex	rat	↑	hippocampus	A sustained upregulation of Prdx6 expression may help protect hippocampal neurons from oxidative stress in a rat model of stroke (localized heat-induced brain injury in the left anterior cortical tectum).	[75]
MCAO	rat	↑	peri-infarct cortex	Prdx6 is involved in the inhibition of curcumin-induced oxidative stress during I/R, and the upregulation of Prdx6 by curcumin attenuates ischemic oxidative damage via SP1 in post-stroke rats.	[76]
MCAO	rat	↓	cerebral cortex	In ischemic brain injury, Prdx6 was increased with a melatonin treatment to protect neuronal cells from ischemic damage.	[78]
MCAO	rat	↑	cortex	Prdx6 may be an important target for immunomodulation and neuroinflammation after ischemic stroke. Prdx6 released from ischemic cells acts as an endogenous ligand for TLR4 and initiates destructive immune responses in ischemic brains.	[79]
OGD/R and MCAO	rat	↑	cortex	The aiPLA2 activity of Prdx6 may play a key role in cerebral ischemia/reperfusion injury by regulating TLR2/4, which induces the production of NF-κB, iNOS, and COX-2, and promote neuroinflammation.	[80]
MCAO	rat	↑	cerebral cortex	Prdx6 knockdown exacerbates cerebral ischemia-reperfusion injury by enhancing PINK1/PARKIN pathway-mediated mitophagy, an effect that increases neuronal apoptosis.	[81]
OGD/R and MCAO	rat	↑	cortex	Prdx6 is upregulated by 4-HBA to protect neurons from cerebral ischemic injury, possibly through the PI3K/Akt pathway.	[83]
Cutting the inner capsule of pig tissue	pig	↑	N/A	Prdx6 is involved in neuroprotective mechanisms after stroke, such as compensating for oxidative stress.	[72]

↑, upregulation; ↓, downregulation; N/A, not applicable; 4-HBA, 4-hydroxybenzyl alcohol; OGD/R, oxygen–glucose deprivation followed by reperfusion; MCAO, middle cerebral artery occlusion; I/R, ischemia/reperfusion; TET-1, endothelial cells overexpressing endothelin-1.
